# Awareness and use of e-cigarettes among urban residents in China

**DOI:** 10.18332/tid/109904

**Published:** 2019-07-02

**Authors:** Luhua Zhao, Lazarous Mbulo, Krishna Palipudi, Jijiang Wang, Brian King

**Affiliations:** 1 Centers for Disease Control and Prevention, Atlanta, United States; 2 University of California San Diego, La Jolla, United States

**Keywords:** adults, China, awareness, e-cigarette, urban

## Abstract

**INTRODUCTION:**

The long-term health effects of e-cigarettes are uncertain, and data on e-cigarette use among Chinese adults are limited. This study examined the prevalence and correlates of e-cigarette awareness and use among urban residents in China. Data came from the China City Adult Tobacco Survey (CCATS), a city-representative household survey conducted using electronic tablets during 2013–2014 in 14 major Chinese cities.

**METHODS:**

CCATS used multistage geographically clustered samples with standardized survey protocols and questionnaire to ensure data comparability. Overall, 31151 adults completed the survey, with sample size varying from 1977 to 3838 across cities, and survey response rates ranging from 79.8% to 97.5%. Respondents were considered current e-cigarette users if they self-reported using e-cigarettes ‘daily’ or ‘less than daily’ at the time of the survey. Descriptive statistics and multivariate logistic regression were conducted. Assessed correlates included: age, education, quit attempts in past 12 months, cigarettes smoked per day, and monthly expenditures on cigarettes.

**RESULTS:**

Overall, 46.7% of respondents were aware of e-cigarettes, 2.9% ever used, and 0.8% currently used. Most current e-cigarette users (93.0%) also currently smoked tobacco. Among male current tobacco smokers, adjusted odds ratio (AOR) of current e-cigarette use was higher among those aged 15–29 (AOR=2.5; 95% CI: 1.5–4.3) or 30–49 (AOR=1.9; 95% CI: 1.0–3.4) than those ≥50 years; those who attempted to quit in the past 12 months than those who did not (AOR=4.7; 95% CI: 2.2–10.1); those with a college degree (AOR=3.4; 95% CI: 1.9–6.2) or just finished high school (AOR=2.2; 95% CI: 1.2–4.2) than those who did not finish high school; and those who smoked ≥15 cigarettes per day (AOR=2.8; 95% CI: 1.4–5.6) than those who smoked fewer.

**CONCLUSIONS:**

These findings reveal that during 2013–2014, many urban Chinese adults were aware of e-cigarettes, while use was relatively low and most current users also smoked tobacco. Continued monitoring of e-cigarettes could help inform public health policy, planning, and practice.

## INTRODUCTION

Electronic cigarettes, or e-cigarettes, are devices designed to deliver nicotine and other additives in the form of an aerosol to the user^1^. This is in contrast to conventional cigarettes, which deliver nicotine and other harmful ingredients to the user in the form of smoke that is created via combustion. Initially developed in the early 2000s, the popularity of modern e-cigarettes has proliferated globally, particularly in some high-income countries, including the United Kingdom, United States, and many countries in the European Union (EU)^1-4^. Overall, there has been a steady decline globally in cigarette smoking over the past decade, particularly in America and Europe^5^. However, in recent years, North American and European countries have experienced a substantial increase in e-cigarette use, particularly among youth^1,6^. It has been estimated that the global e-cigarette market could reach more than US$20 billion by 2022, with a forecasted increase of 17% annually^7^.

To date, the long-term effects of e-cigarettes on individual and population-level health remain uncertain^1^. Evidence has shown that e-cigarettes could have potential benefits to adult smokers who are not pregnant, if used as a complete substitute for regular cigarettes and other smoked tobacco products^8,9^. However, the evidence is insufficient to recommend e-cigarettes for smoking cessation in adults including pregnant women^8,10-12^. Moreover, e-cigarettes are not safe, particularly for youth, young adults, pregnant women, and adults who do not currently use tobacco products^1^. Most e-cigarettes contain nicotine. Nicotine is highly addictive, can harm adolescent brain development, which continues into the early to mid-20s, and is toxic to developing fetuses^1^. In addition, a growing body of scientific evidence from numerous countries indicates that e-cigarette use may lead to future cigarette smoking among youth^1,13^.

Research indicates that e-cigarette aerosol generally contains fewer toxic chemicals than the deadly mix of 7000 chemicals in smoke from regular cigarettes^1^. However, e-cigarette aerosol is not harmless. It can contain harmful and potentially harmful substances besides nicotine, including heavy metals like lead, volatile organic compounds, and carcinogens^1,13^. The potential population-level risks of these products are compounded by the manner in which they are advertised and promoted, particularly in ways that may influence initiation among youth and young adults. Specifically, the 2016 U.S. Surgeon General’s Report concluded that e-cigarettes are marketed by promoting flavors and using a wide variety of media channels and approaches that have been used in the past for marketing conventional tobacco products to youth and young adults^1,13^.

The diversification of the tobacco product landscape in recent years has warranted the updating of existing tobacco control policies to include the variety of products being used among adults and youth, including e-cigarettes^1^. However, given the dynamic and evolving e-cigarette landscape^1^, including variations in the rise of e-cigarette use across countries, population-based policies related to e-cigarette use such as regulation of the manufacturing, marketing, sale, and use of these products, have varied across countries. E-cigarettes are currently regulated at various levels in 83 countries^14^. For China, two Chinese government agencies issued a joint directive in September 2018 to prohibit e-cigarette sales to minors^15^. Despite being the major producer of e-cigarettes globally^16^, the e-cigarette market in China is relatively small, with recent estimates of US$175 million in 2016^7,16^. Conventional cigarettes still dominate the tobacco product market in China, with an estimated 28.1% and 26.6% of Chinese adults being current cigarette smokers in 2009 and 2018, respectively^17,18^.

Continuous monitoring of e-cigarette use among different population groups is important to guide the development and implementation of policies to maximize any potential benefits of e-cigarettes, while minimizing potential risks at the population level, particularly with regard to vulnerable populations such as youth and young adults^1^. Internationally, assessments of e-cigarette awareness and use are growing. For example, findings from the International Tobacco Control Survey (ITC) during 2009–2013 found that ever use of e-cigarettes among current and ever adult smokers across 10 countries ranged from 2% in China to 20% in Australia, while current use ranged from 0.05% in China to 14% in Malaysia^19^. In China, systematic surveillance data on e-cigarettes remain particularly limited. Among youth, a national survey conducted in 2013–14 indicated 45.0% of middle school students were aware of e-cigarettes and 1.2% reported e-cigarette use in the past 30 days^20^. The China ITC study in 2009 found that an estimated 31.0% of Chinese adults were aware of e-cigarettes and 2% had ever tried e-cigarettes^19^. A subsequent study from the 2013–2014 China City Adult Tobacco Survey (CCATS) found that e-cigarette awareness and use were relatively low across 14 individual cities throughout China^21^. However, few published studies have systematically assessed general awareness and use of e-cigarettes among adults in China in recent years, or variations in these measures across population groups, including sociodemographic characteristics and smoking behaviors.

CCATS provides a unique opportunity to examine and describe patterns of awareness, ever use, and current use of e-cigarettes in China to address an existing gap in the scientific literature. Specifically, this study examined the prevalence and correlates of e-cigarette awareness and e-cigarette use among adults in 14 major Chinese cities using combined city data from CCATS. As cities are considered the centers of social and economic development in China, tobacco control efforts in urban areas can be particularly influential for surrounding areas and serve as examples for national efforts^22^.

## METHODS

### Data source

CCATS is a city-representative, household-based, cross-sectional survey of non-institutionalized adults aged 15 years or older, which was conducted during 2013–2014 in 14 major Chinese cities including Anshan, Beijing, Changchun, Haerbin, Hangzhou, Kelamayi, Lanzhou, Luoyang, Nanchang, Qingdao, Shenyang, Shenzhen, Tangshan, and Tianjin^21^. Across the 14 cities, CCATS used a multistage geographically clustered sample design with standardized survey protocols and questionnaire to ensure data comparability. Data were collected using electronic tablets. A roster of eligible adults living in the household was obtained from interviews with adults aged ≥18 years. Minors between 15 and 18 years of age were allowed to provide household information only if there was no one aged ≥18 years in the household. However, any persons aged ≥15 years in the household were considered adults and could potentially be selected to participate in the survey. The estimated target population across cities ranged from 0.26 million to 14.3 million, with an estimated total of 52.8 million adults aged 15 years or older in 14 cities. Overall, 31151 adults completed the survey, including 15008 males and 16143 females. The overall sample size varied from 1977 to 3838 across cities. The overall survey response rate ranged from 79.8% to 97.5% across 14 cities. The overall prevalence of current smokers was 21.1%; with prevalence 39.1% among males and 2.1% among females.

### Measures

#### Awareness of e-cigarettes

Awareness of e-cigarettes was defined as a response of ‘yes’ to the question: ‘Have you ever heard of electronic cigarettes? Electronic cigarettes are electronic inhalers that vaporize nicotine in liquid solution into an aerosol mist, simulating the act of tobacco smoking’.

#### Ever use of e-cigarettes

Ever use of e-cigarettes was defined as a response of ‘yes’ to the question: ‘Have you ever, even once, used an electronic cigarette?’.

#### Current use of e-cigarettes

Current use of e-cigarettes was defined as a response of ‘daily’ or ‘less than daily’ to the question: ‘Do you currently use electronic cigarettes on a daily basis, less than daily, or not at all?’.

#### Smoking behaviors

Current combustible tobacco product smoking was defined as a response of ‘daily’ or ‘less than daily’ to the question: ‘Do you currently smoke tobacco on a daily basis, less than daily, or not at all?’. Hereafter, smokers of combustible tobacco products are referred to as tobacco smokers. Former tobacco smokers and never tobacco smokers were defined using the aforementioned question in combination with a second question: ‘In the past, have you smoked tobacco on a daily basis, less than daily or not at all?’. Former tobacco smoking was defined as a response of ‘not at all’ to the first question and ‘daily’ or ‘less than daily’ to the second question. Never tobacco smoking was defined as a response of ‘not at all’ to both questions.

For the purposes of this study, cigarette (manufactured) consumption per day (CPD) was calculated among current tobacco smokers using answers from the question: ‘On average, how many of manufactured cigarettes do you currently smoke each day?’. For those who smoked less than daily, a further question was asked: ‘On average, how many manufactured cigarettes do you currently smoke each week?’. The weekly consumption reported for that question was then divided by seven to determine CPD.

Monthly cost spent on manufactured cigarettes was calculated using the unit cost of one manufactured cigarette obtained from the respondent’s last purchase, and CPD as defined above. A cut-off point of 15 CPD (55th percentile) was chosen to divide respondents into two comparable percentile groups, including one high-consumption group and one low-consumption group; this cut-off point was selected because it was the integer closest to the mean (15.1).

Quit attempts in the last 12 months was assessed using the question: ‘During the past 12 months, have you tried to stop smoking?’. Respondents who indicated ‘yes’ were considered to have made a quit attempt.

#### Sociodemographic characteristics

Assessed sociodemographic characteristics included: sex, age group (15–29, 30–49, and ≥50 years), educational attainment (less than high school, high school graduate, college or above), smoking status (current tobacco smoker, former tobacco smoker, never tobacco smoker), and city-level disposable annual income in Chinese Renminbi (RMB) (less than 28000 and ≥28000 RMB). The 28000 RMB equal US$4563 using the average conversion rate for July 2013 (1 RMB = 0.162995 US$)^23^. The threshold of 28000 RMB was chosen so that the 14 cities could be divided into two groups for comparison and ease of interpretation, with one high-income group and one low-income group. City-level disposable income was used because individual income of the survey respondents was not included in the questionnaire.

### Analysis

Descriptive statistics, including point estimates and 95% confidence intervals (CI), were calculated for e-cigarette awareness, ever use, and current use; estimates were calculated overall and by smoking behaviors and sociodemographic characteristics.

Multivariate logistic regressions were performed to examine correlates of current e-cigarette use; adjusted odds ratios (AOR) and corresponding 95% CI were assessed. Assessed correlates included: sex, age, education, and city-level average disposable income.

Given that most current e-cigarette users were male current tobacco smokers (88.7%) and the sample size for female current tobacco smokers was limited (<20), further analyses of correlates focused on current male tobacco smokers. Among these respondents, quit attempts in the past 12 months, cigarettes smoked per day, and monthly expenditures on cigarettes were also assessed. City was initially included in the model, but was later dropped as its inclusion did not significantly modify the results, and variations between cities was not a primary focus of this study.

SAS (Ver. 9.4) was used for data processing and SAS-Callable SUDAAN (Ver. 11.0) was used for analyses. All data were weighted to the estimated target population individually in all 14 cities^21^. The weighting process used for CCATS was comparable to the process used for the Global Adult Tobacco Survey, which has been discussed in detail elsewhere^24^. Due to limited sample size for certain variables, a supplemental analysis was performed to confirm the validity of the employed model. Specifically, STATA firthlogit procedure was used to run penalized logistic regression, which is generally more robust when analyzing sparse data. The AOR estimates were comparable to those obtained via SUDAAN, thus reinforcing the validity of the multivariate logistic regression approach used in this study.

## RESULTS

### E-cigarette awareness

Among adults in all 14 assessed cities, 46.7% were aware of e-cigarettes (Table 1). Awareness was 54.9% among males and 38.2% among females. By smoking status, awareness was 66.3% among current tobacco smokers, 54.8% among former tobacco smokers, and 40.8% among never tobacco smokers. Awareness was 49.6% among those aged 15–29 years, 52.9% among those aged 30–49 years, and 35.6% among those aged 50 years and older. Awareness was 33.9% among those with less than high school education, 46.9% among those with a high school education, and 54.9% among those with a college or above education.

**Table 1:** Weighted percentage and adjusted odds ratio of awareness, ever use, and current use of e-cigarettes among adults aged 15 years or older, by selected sociodemographic characteristics, China City Adult Tobacco Survey, 2013–2014

*Sociodemographic characteristics*	*Sample size*	*Awareness of e-cigarettes^a^*		*Ever used e-cigarettes^b^*		*Current use e-cigarettes^c^*	

		*Percentage*	*AOR (95% CI)*	*Percentage*	*AOR (95% CI)*	*Percentage*	*AOR (95% CI)*
**Overall**	31151	46.7 (44.5 – 49.0)	–	2.9 (2.5 – 3.3)	–	0.8 (0.6 – 1.1)	–
**Gender**							
Male	15008	54.9 (52.2 – 57.6)	**1.3 (1.2–1.5)**	5.0 (4.3 – 5.8)	1.4 (0.9–2.2)	1.5 (1.0 – 2.1)	0.8 (0.4–1.9)
Female	16143	38.2 (35.8 – 40.6)	Referent	0.6 (0.4 – 1.0)	Referent	0.1 (0.1 – 0.2)	Referent
**Smoking status**							
Current	6756	66.3 (63.5 – 69.0)	**2.8 (2.5–3.2)**	10.9 (9.5 – 12.4)	**18.5 (11.8–28.9)**	3.6 (2.6 – 4.9)	**78.5 (28.0–219.5)**
Former	1698	54.8 (50.0 – 59.5)	**2.1 (1.6–2.6)**	3.3 (2.2 – 4.9)	**5.6 (3.3–9.5)**	0.3 (0.1 – 0.8)	**8.0 (1.9–33.7)**
Never	22697	40.8 (38.4 – 43.2)	Referent	0.6 (0.4 – 0.9)	Referent	0.1 (0.0 – 0.1)	Referent
**Age**							
15–29	4912	49.6 (45.9 – 53.3)	**1.6 (1.4–1.9)**	2.7 (2.1 – 3.5)	1.4 (1.0–1.9)	0.8 (0.5 – 1.3)	**2.1 (1.3–3.6)**
30–49	11866	52.9 (50.2 – 55.6)	**1.7 (1.5–1.9)**	3.4 (2.7 – 4.2)	1.2 (0.9–1.7)	1.0 (0.6 – 1.7)	**1.8 (1.0–3.2)**
≥50 years	14373	35.6 (33.6 – 37.8)	Referent	2.4 (1.9 – 2.9)	Referent	0.5 (0.3 – 0.7)	Referent
**Education level**							
Less than high school	11251	33.9 (31.5 – 36.3)	Referent	2.0 (1.7 – 2.4)	Referent	0.4 (0.3 – 0.6)	Referent
High school	9182	46.9 (44.2 – 49.6)	**1.6 (1.4–1.8)**	2.9 (2.3 – 3.6)	**1.5 (1.1–2.0)**	0.9 (0.5 – 1.6)	**1.9 (1.1–3.5)**
College or above	10549	54.9 (51.5 – 58.3)	**2.3 (1.9–2.6)**	3.4 (2.8 – 4.2)	**2.1 (1.5–2.8)**	1.0 (0.7 – 1.5)	**2.5 (1.4–4.3)**
**Disposable annual income**							
<28000	17266	49.1 (45.5 – 52.8)	**1.3 (1.1–1.5)**	3.0 (2.5 – 3.5)	1.1 (0.8–1.4)	0.9 (0.7 – 1.2)	1.2 (0.7–2.1)
≥28000 RMB (US$4563)^d^	13885	46.0 (43.2 – 48.7)	Referent	2.8 (2.3 – 3.5)	Referent	0.8 (0.5 – 1.2)	Referent

AOR: adjusted odds ratio, CI: confidence interval. Statistically significant odds ratios noted in bold.

a Awareness of e-cigarettes was defined as a response of ‘yes’ to the question: ‘Have you ever heard of electronic cigarettes? Electronic cigarettes are electronic inhalers that vaporize nicotine in liquid solution into an aerosol mist, simulating the act of tobacco smoking’.

b Ever use of e-cigarettes was defined as a response of ‘yes’ to the question: ‘Have you ever, even once, used an electronic cigarette?’.

c Current use of e-cigarettes was defined as a response of ‘daily’ or ‘less than daily’ to the question: ‘Do you currently use electronic cigarettes on a daily basis, less than daily, or not at all?’.

d Conversion rate to US$ was calculated based on average conversion rate from July 2013.

Last Accessed on 20 February 2019 from https://www.x-rates.com/average/?from=CNY&to=USD&amount=1&year=2013.

The adjusted odds ratio of being aware of e-cigarettes was higher among: males (AOR=1.3; 95% CI: 1.2–1.5) than females; current tobacco smokers (AOR=2.8; 95% CI: 2.5–3.2) and former tobacco smokers (AOR=2.1; 95% CI: 1.6–2.6) than never smokers; those aged 15–29 (AOR=1.6; 95% CI: 1.4–1.9) or 30–49 (AOR=1.7; 95% CI: 1.5–1.9) than those aged 50 years or older; and those who just finished high school or had a college or greater education (AOR=1.6; 95% CI: 1.4–1.8 and AOR=2.3; 95% CI: 1.9–2.6, respectively) than those who had a less than high school education (Table 1).

### Ever e-cigarette use

The prevalence of ever e-cigarette use among all adults in the 14 cities was 2.9% overall, 5.0% among males and 0.6% among females (Table 1). By smoking status, ever use was 10.9% among current tobacco smokers, 3.3% among former tobacco smokers, and 0.6% among never tobacco smokers. By age group, ever use of e-cigarettes was 2.7% among those aged 15–29 years, 3.4% among those aged 30–49 years, and 2.4% among those aged ≥50 years. Ever use was 2.0% among those with less than a high school education, 2.9% among those with a high school education, and 3.4% among those with a college or greater education.

The adjusted odds ratio of having ever used e-cigarettes was greater among: current tobacco smokers (AOR=18.5; 95% CI: 11.8–28.9) and former smokers (AOR=5.6; 95% CI: 3.3–9.5) than never smokers; those aged 15–29 years (AOR=1.4; 95% CI: 1.0–1.9) than those aged ≥50 years; and those who just finished high school or had a college or greater education (AOR=1.5; 95% CI: 1.1–2.0 and AOR=2.1; 95% CI: 1.5–2.8, respectively) than those who had a less than high school education (Table 1).

### Current e-cigarette use

The prevalence of current e-cigarette use was 0.8% overall, 1.5% among males and 0.1% among females (Table 1). By smoking status, current e-cigarette use was 3.6% among current tobacco smokers, 0.3% among former tobacco smokers, and 0.1% among never smokers. Current e-cigarette use was 0.5% among those aged ≥50 years or older, 0.8% among those aged 15–29 years, and 1.0% among those aged 30–49 years. Current e-cigarette use was 0.4% among those with less than a high school education, 0.9% among those with a high school education, and 1.0% among those with a college degree or higher education.

The adjusted odds ratio of current e-cigarette use was greater among: current tobacco smokers (AOR=78.5; 95% CI: 28.0–219.5) and former smokers (AOR=8.0; 95% CI: 1.9–33.7) than never smokers; those aged 15–29 years (AOR=2.1; 95% CI: 1.3– 3.6) and 30–49 years (AOR=1.8; 95% CI: 1.0–3.2) than those aged 50 years or older; and those who just finished high school or had a college or greater education (AOR=1.9; 95% CI: 1.1–3.5 and AOR=2.5; 95% CI: 1.4–4.3, respectively) compared to those who had a less than high school education (Table 1).

**Figure 1:**
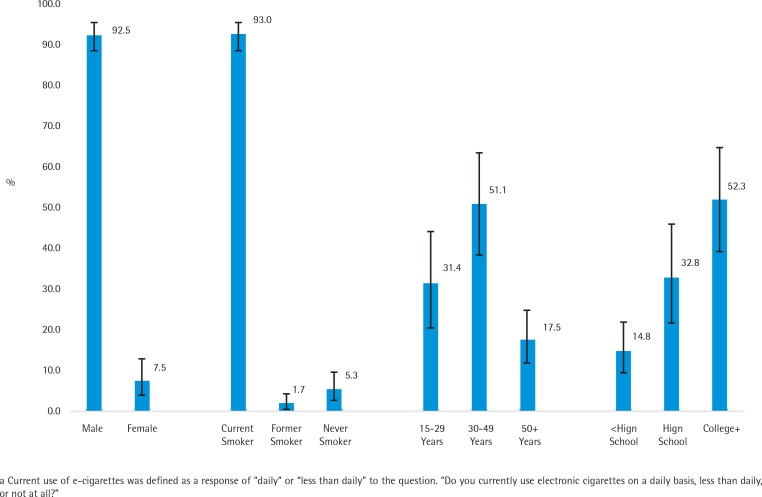
Percentage distribution of current e-cigarette users^a^ 15 years or older by selected demographic characteristics - China City Adult Tobacco Survey, 2013-14.

Among current e-cigarette users, most (92.5%) were male (Figure 1). Most (93.0%) current e-cigarette users were also current tobacco smokers: 5.3% were never smokers and 1.7% were former smokers. By age, 31.4% of current e-cigarette users were aged 15–29 years, 51.1% were aged 30–49 years, and 17.5% were aged ≥50 years or older. By education, 52.3% of current e-cigarette users had a college degree, 32.8% graduated from high school, and 14.8% had less than a high school education.

Among male current tobacco smokers who were also current e-cigarette users (Table 2), 33.9% were aged 15–29, 51.3% were aged 30–49, and 14.8% were aged 50 years or older. By education, 13.6% had a less than a high school education, 31.6% had a high school education, and 54.7% had a college degree. Approximately two-thirds (68.7%) of them smoked 15 or more cigarettes per day, and approximately half (55.1%) tried to quit smoking in the past 12 months. Approximately one-quarter (24.7%) had an annual disposable income <28000 RMB (US$ 4536).

**Table 2:** Weighted percentage and adjusted logistic regression among current male tobacco smokers^a^ on current e-cigarette use^b^, by selected sociodemographic characters, China City Adult Tobacco Survey, 2013–14

*Independent variables*	*Sample size*	*Percentage (95% CI)*	*AOR (95% CI)*
**Age**			
15–29	33	33.9 (22.4–47.6)	**2.5 (1.5–4.3)**
30–49	94	51.3 (38.6–63.9)	**1.9 (1.0–3.4)**
≥50 years	55	14.8 (9.7–21.9)	Referent
**Education level**			
Less than high school	40	13.6 (8.7–20.7)	Referent
High school	58	31.6 (20.7–45.1)	**2.2 (1.2–4.2)**
College or above	84	54.7 (41.0–67.8)	**3.4 (1.9–6.2)**
**Cigarette consumption per day**			
1–14	66	31.3 (21.7–42.8)	Referent
≥15	96	68.7 (57.2–78.3)	**2.8 (1.4–5.6)**
**Tried to quit in past 12 months**			
Yes	90	55.1 (40.8–68.5)	**4.7 (2.2–10.1)**
No	92	44.9 (31.5–59.2)	Referent
**Monthly cost on cigarettes**			
<150	39	20.2 (13.7–28.8)	0.7 (0.3–1.6)
150–250	46	26.9 (18.9–36.8)	0.9 (0.5–1.7)
250–450	42	23.2 (16.5–31.6)	0.8 (0.4–1.6)
≥450 RMB	42	29.7 (20.9–40.2)	Referent
**Disposable annual income**			
<28000	109	24.7 (15.3–37.2)	1.2 (0.6–2.3)
≥28000 RMB (US$ 4563)^c^	73	75.3 (62.8–84.7)	Referent

AOR: adjusted odds ratio, CI: confidence interval. Statistically significant odds ratios noted in bold.

a Current tobacco smokers were defined as respondents who indicated a response of ‘daily’ or ‘less than daily’ to the question: ‘Do you currently smoke tobacco on a daily basis, less than daily, or not at all?’.

b Current use of e-cigarettes was defined as a response of ‘daily’ or ‘less than daily’ to the question: ‘Do you currently use electronic cigarettes on a daily basis, less than daily, or not at all? Electronic cigarettes are electronic inhalers that vaporize nicotine in liquid solution into an aerosol mist, simulating the act of tobacco smoking’.

c Conversion rate to US$ was calculated based on average conversion rate from July 2013.

Last Accessed on 20 February 2019 from https://www.x-rates.com/average/?from=CNY&to=USD&amount=1&year=2013.

The adjusted odds ratio of current e-cigarette use among current male smokers was significantly higher among those aged 15–29 years (AOR=2.5; 95% CI: 1.5–4.3) or 30–49 years (AOR=1.9; 95% CI: 1.0–3.4) than those aged ≥50 years; among those who attempted to quit than those who made no quit attempt in the past 12 months (AOR=4.7; 95% CI: 2.2–10.1); among those with a high school or college or greater education, (AOR=2.2; 95% CI: 1.2–4.2 and AOR=3.4; 95% CI: 1.9–6.2, respectively), than those with less than high school education; and those who smoked ≥15 cigarettes per day (AOR=2.8; 95% CI: 1.4–5.6) than those who smoked 1–14 cigarettes per day. No significant variation in odds was observed in either monthly expenditures on cigarettes or disposable income (Table 2).

## DISCUSSION

The findings from this study reveal that approximately half of urban adult residents in 14 Chinese cities were aware of e-cigarettes, while 2.9% had ever used e-cigarettes, and 0.8% currently used e-cigarettes. Current e-cigarette use was higher among current and former smokers of combustible tobacco, younger persons, and those with a college or greater education. Additionally, most current e-cigarette users were also current tobacco smokers. Given that the tobacco product landscape continues to diversify, and the use of e-cigarettes has increased in several countries in recent years, these findings could provide a useful baseline measure of e-cigarette related behaviors among urban Chinese adults. Continued monitoring of e-cigarette use in China could, therefore, help inform public health policy, planning, and practice.

In the present study, awareness of e-cigarettes among current tobacco smokers was 66.3%, which fell within the range observed nationally in the US (76.9%) and EU countries during the same time period, including Sweden (57.1%), Belgium (69.3%), Ireland (66.6%), and Lithuania (65.8%)^3,25^. Variations in awareness of e-cigarettes could be the result of multiple social, cultural, and economic factors. One of these factors is advertising and promotion^1^. E-cigarettes have been widely advertised since mid-2000^26^. This has likely contributed to the relatively prominent levels of awareness of these products in China and other countries.

The prevalence of having ever used e-cigarettes also varied in urban China compared to the EU countries during the same time period. Although the overall prevalence of ever e-cigarette use was 2.9%, prevalence was particularly high among current tobacco smokers (10.9%). This level of ever use among current tobacco smokers was lower than the US estimate (36.5%) from 2013, but comparable to national data in the EU collected in 2012, specifically in Belgium (11.5%), Ireland (12.1%) and Lithuania (11.8%), as well as Italy (8.8%), Spain (10.9%), and Slovakia (7.9%)^3,25^. Ever use was also particularly high among former smokers and males. Ever use of e-cigarettes, including experimentation of these products, could be influenced by multiple factors, such as curiosity, the belief that these products could help with tobacco cessation, and marketing by manufacturers^1^. Additionally, in China, the relatively lower rates of use could be because conventional cigarettes cost as low as 3 RMB per pack (less than US$0.50)^27^, and that using e-cigarettes requires a relatively high initial investment for e-cigarette starter kits^7,16^.

Although current e-cigarette use was relatively low at 0.8% in the present study, it was higher than the 0.05% reported by ITC^19^. The difference is likely due to the 4-year period between surveys, and the factors that could influence e-cigarette ever use noted above. Variations in current e-cigarette use were observed in the present study across population groups. The disparity between males (1.5%) and females (0.1%) mirrors the disparity in prevalence of current cigarette smoking between males (39.1%) and females (2.1%) from CCATS; this disparity is likely due to the longstanding social unacceptability of female tobacco product use in Chinese society. Disparities in use were also documented by educational status; specifically, e-cigarette use, as well as awareness, was higher among those with more education, which could reflect the relatively high initial cost of e-cigarettes relative to cigarettes, as well as the manner in which the products are advertised and promoted^7^. Most notably, tobacco smokers were significantly more likely to be current e-cigarette users. The results were consistent with previous research from European and North American countries^28-29^. In addition, high nicotine dependency, measured as smoking 15 or more cigarettes per day, was a significant correlate of current e-cigarette use in the present study. It is possible that many of the current smokers in this study may have been using e-cigarettes in an attempt to quit smoking conventional cigarettes. E-cigarettes have been advertised as a smoking cessation aid^26^. However, existing science on the effectiveness of e-cigarettes for the purposes of smoking cessation is inconclusive, and findings from high-income countries suggest that a majority of adult e-cigarette users continue to smoke conventional cigarettes^11,28-30^. Similarly, in the present study, more than 90% of current e-cigarette users were current tobacco smokers. E-cigarettes have the potential to benefit adult smokers who are not pregnant if used as a complete substitute for regular cigarettes and other smoked tobacco products^1,8^. However, the continued use of both e-cigarettes and conventional cigarettes is not an effective way to safeguard health given that smoking even a few cigarettes per day carries significant health risks^8^. Additionally, research indicates that dual users of e-cigarettes and cigarettes have comparable, and in some cases higher, toxicant levels in their body compared to exclusive cigarette smokers; therefore, transitioning completely to e-cigarettes from conventional cigarettes would be required for a meaningful health benefit to occur^31^.

Population-based policies related to e-cigarette use, including regulation of the manufacturing, marketing, sale, and use of these products, may help minimize potential health risks of these products at the population level, particularly among youth and young adults^1^. In China, the manufacturing, sales, advertising, and use of e-cigarettes is still largely unregulated^14^, except prohibitions on the sale of these products to minors^15^. Although use of e-cigarettes is still relatively low in China, especially compared to conventional cigarettes, the marked increase in use that has occurred among youth in other countries such as the US could pose great challenges to tobacco control efforts in China given that traditional tobacco control measures at present focus on combustible tobacco products, not e-cigarettes^32^. Moreover, available research suggests that e-cigarette use among youth could potentially lead to future use of conventional tobacco products^13^. Additionally, a recent study suggested that among Chinese middle school students who never smoked conventional cigarettes, e-cigarette users were more likely than non-users to state that they would enjoy cigarette smoking and to show interest in trying conventional cigarettes in the near future^20^. The situation is compounded by the prominent promotion and advertising of these products, which could be targeted towards and appeal to young people both in China and other countries^33,34^. The World Health Organization notes the importance of preventing the initiation of e-cigarettes among youth and non-users, as well as preventing the tobacco industry’s interference in tobacco control activities^35^. The implementation of a comprehensive tobacco control strategy that addresses all forms of tobacco products used, including e-cigarettes, is therefore important to minimize population-level risks of e-cigarettes, particularly among vulnerable populations^1^.

### Limitations

This study has some limitations. First, the study used self-reported data, which could lead to underestimation of actual e-cigarette use due to recall bias. However, previous research suggests that self-reports of conventional tobacco use are generally valid^36,37^; thus, self-reported estimates of emerging tobacco products such as e-cigarettes are likely similarly valid. Second, the study collected data in 14 cities sampled from different regions across China. Therefore, although the estimates reflect e-cigarette use in urban areas in China, the findings are not necessarily generalizable to the broader population of urban Chinese residents, or the country as a whole. Third, the study was a descriptive analysis of awareness and use, and it was not possible to assess underlying factors that may have contributed to the perception and use of e-cigarettes, including why and how e-cigarettes were used across different groups. Fourth, participants <25 years old were included in assessments of education to ensure a robust sample size for the analysis; this could introduce bias given that these respondents were not yet of sufficient age to attain the full scope of education categories assessed. Fifth, sample size was limited for certain analyses, which introduced large standard errors and did not allow for the presentation of nuanced analyses among certain population groups (e.g. women). Sixth, the definition of current use of cigarettes, current use of e-cigarettes, and quit attempt in this study may be different from other research; thus, comparability may be limited. Moreover, for certain indicators (e.g. odds ratios related to current tobacco smoking), standard errors were large due to limited sample size, and thus, should be interpreted with caution. However, the majority of estimates could be presented and only a small minority of estimates were suppressed due to limited relative standard errors. Nonetheless, continued surveillance of these populations is warranted, as the e-cigarette landscape continues to diversify and use may potentially increase among these populations. Finally, constructs such as awareness and use are highly sensitive to changes over time. Given that the data for this study were from 2013–14, they may not reflect more recent patterns. However, although the constructs are sensitive to change, estimates of use in other countries have been fairly stable since 2013–2014 (e.g. United States)^13^, suggesting that estimates may not deviate markedly from 2013–2014 in China. Nonetheless, these data are still useful for informing public health policy, planning, and practice.

## CONCLUSIONS

In 14 Chinese cities during 2013–2014, about half of adults were aware of e-cigarettes, while use of these products remained relatively low. However, marked variations in awareness and use of e-cigarettes were apparent across population groups, with prevalence of use being particularly high among current tobacco smokers. Continued monitoring of e-cigarette awareness and use is important to inform the regulation of e-cigarettes and the implementation of proven population-based strategies to maximize any potential benefits of e-cigarettes among adult smokers while minimizing established risks at the population level, particularly among young people. Additionally, more nuanced measures on e-cigarette use behaviors, including characteristics of and reasons for use, could be especially informative. Further research on the appeal of different e-cigarette products, and their impact on smokers, could also help inform public health policy and practice in China.

## References

[1] U.S. Department of Health and Human Services (2016). E-Cigarette Use Among Youth and Young Adults: A Report of the Surgeon General.

[2] Cobb NK, Byron MJ, Abrams DB, Shields PG (2010). Novel nicotine delivery systems and public health: The rise of the ‘e-cigarette’. Am J of Public Health.

[3] Vardavas CI, Filippidis FT, Agaku IT (2015). Determinants and prevalence of e-cigarette use throughout the European Union: a secondary analysis of 26, 566 youth and adults from 27 countries. Tob Control.

[4] Filippidis FT, Laverty AA, Gerovasili V, Vardavas CI (2017). Two-year trends and predictors of e-cigarette use in 27 European Union member states. Tob Control.

[5] Burki TK (2018). Big tobacco turns its attention to Africa. Lancet Respir Med.

[6] Yoong SL, Stockings E, Chai LK (2018). Prevalence of electronic nicotine delivery systems (ENDS) use among youth globally: a systematic review and meta-analysis of country level data. Aust N Z J Public Health.

[7] Digital Trends Puff, Puff, recharge. E-cigarettes are booming, and China is ground zero.

[8] U.S. Department of Health and Human Services (2014). The Health Consequences of Smoking – 50 Years of Progress: A Report of the Surgeon General. U.S.

[9] Tatullo M, Gentile S, Paduano F, Santacroce L, Marrelli M (2016). Crosstalk between oral and general health status in e-smokers. Medicine.

[10] Hartmann-Boyce J, McRobbie H, Bullen C, Begh R, Stead L, Hajek P (2016). Electronic cigarettes for smoking cessation. Cochrane Database Syst Rev.

[11] U.S. Preventive Services Task Force Final Recommendation Statement: Tobacco Smoking Cessation in Adults, Including Pregnant Women: Behavioral and Pharmacotherapy Interventions.

[12] Glantz S, Bareham D (2018). E-Cigarettes: Use, Effects on Smoking, Risks, and Policy Implications. Annu Rev Public Health.

[13] National Academies of Sciences, Engineering, and Medicine (2018). Public health consequences of E-Cigarettes.

[14] Institute for Global Tobacco Control Country Laws Regulating E-cigarettes: A Policy Scan.

[15] Two Agencies Issued Directive Prohibiting E-cigarette Sales to Minors.

[16] Business Wire China E-Cigarettes Market Report 2017 - A Market With Massive Potential But Unusual Challenges - Research and Markets.

[17] Yang GH (2011). Global Adults Tobacco Survey (GATS) China 2010 country report.

[18] Latest Adult Tobacco Survey Results Released.

[19] Gravely S, Fong G, Cummings K (2014). Awareness, Trial, and Current Use of Electronic Cigarettes in 10 Countries: Findings from the ITC Project. Int J Environ Res Public Health.

[20] Xiao L, Parascandola M, Wang C, Jiang Y (2018). Perception and Current Use of E-cigarettes Among Youth in China. Nicotine Tob Res.

[21] Liang XF (2015). Report of China City Adult Tobacco Survey 2013-14: A 14-city experience.

[22] Redmon P, Koplan J, Eriksen M, Li S, Wang K (2014). The role of cities in reducing smoking in China. Int J Environ Res Public Health.

[23] X-Rates: Monthly Average.

[24] Global Adult Tobacco Survey Collaborative Group (2010). Global Adult Tobacco Survey (GATS): Sample Weights Manual, Version 2.0.

[25] King B, Patel R, Nguyen K, Dube S (2015). Trends in awareness and use of electronic cigarettes among US adults, 2010-2013. Nicotine Tob Res.

[26] Xu X, Wang X, Zhang X, Liu Y, He H, Mackay J (2016). The debate on regulation of e-cigarettes in China. Lancet.

[27] Zhang P South China Morning Post. Are cigarettes too affordable for China’s young people?.

[28] Wang M, Wang JW, Cao SS, Wang HQ, Hu RY (2016). Cigarette Smoking and Electronic Cigarettes Use: A Meta-Analysis. Int J Environ Res Public Health.

[29] Levy D, Yuan X, Li Y (2017). The Prevalence and Characteristics of E-Cigarette Users in the U.S. Int J Environ Res Public Health.

[30] Caraballo R, Shafer P, Patel D, Davis K, McAfee T (2017). Quit Methods Used by US Adult Cigarette Smokers, 2014-2016. Prev Chronic Dis.

[31] Goniewicz ML, Smith DM, Edwards KC (2018). Comparison of Nicotine and Toxicant Exposure in Users of Electronic Cigarettes and Combustible Cigarettes. JAMA Netw Open.

[32] Feldman EA, Yue C E-Cigarette Regulation in China: The Road Ahead. Faculty Scholarship.

[33] Jiang N, Ho S, Lam T (2017). Electronic cigarette marketing tactics in mainland China. Tob Control.

[34] Health Line Tobacco Companies Taking Over the E-cigarette Industry.

[35] World Health Organization Electronic Nicotine Delivery Systems and Electronic Non-Nicotine Delivery Systems (ENDS/ENNDS).

[36] Agaku I, King B (2014). Validation of self-reported smokeless tobacco use by measurement of serum cotinine concentration among US adults. Am J Epidemiol.

[37] Caraballo RS, Giovino GA, Pechacek T, Mowery PD (2001). Factors associated with discrepancies between self-reports on cigarette smoking and measured serum cotinine levels among persons aged 17 years or older: Third National Health and Nutrition Examination Survey, 1988-1994. Am J Epidemiol.

